# Epicardial adipose tissue thickness as a potential predictor of gestational diabetes mellitus: a prospective cohort study

**DOI:** 10.1186/s12872-020-01480-7

**Published:** 2020-04-19

**Authors:** Jing Liu, Guang Song, Tao Meng, Ge Zhao

**Affiliations:** 1 Department of Obstetrics, The First Affiliated Hospital of China Medical University, No. 155 Nanjing North Street, Heping District, Shenyang, 110001 Liaoning Province China; 2 Department of Ultrasound, Shengjing Hospital of China Medical University, Shenyang, China

**Keywords:** Gestational diabetes mellitus, Epicardial adipose tissue, Prediction, Echocardiography

## Abstract

**Background:**

Gestational diabetes mellitus (GDM) is the most common metabolic disorder that can occur during pregnancy and is associated with a long-term risk of both maternal and neonatal comorbidities. This study aimed to investigate the association between echocardiographic epicardial adipose tissue (EAT) and the risk for GDM during the early second trimester of pregnancy.

**Method:**

We recruited all singleton pregnancies between January 2014 and December 2018 at 16 weeks + 0 days to 19 weeks + 6 days. We then used generalized linear models to calculate odds ratios (ORs) and 95% confidence intervals (CIs) for EAT as a potential predictor for GDM. Receiver-operating-characteristic (ROC) analysis was then conducted to investigate the discriminative capacity of any individual maternal factor for the prediction of GDM.

**Results:**

In total, our study involved 314 pregnant women with GDM and 1832 pregnant women without GDM. Multivariate regression analysis revealed that EAT thickness (OR = 2.87; 95% CI: 2.49–3.31) was significantly associated with the presence of GDM (*P <* 0.001). Furthermore, EAT thickness was also significantly associated with a range of adverse outcomes in the GDM group, including large size for gestational age, neonatal hypoglycemia, admission to the neonatal intensive care unit, preterm delivery, and hyperbilirubinemia (*P <* 0.001). ROC analysis revealed that the area under the curve was 0.790 (95% CI: 0.768–0.812). When the cutoff value for EAT thickness was set to 5.49 mm, the sensitivity was 95.2% and the specificity was 50.5%.

**Conclusions:**

Echocardiographic EAT thickness is positively and significantly associated with both the risk of GDM and adverse outcomes related to GDM. Echocardiographic EAT has the potential to predict GDM prior to actual clinical diagnosis.

## Background

Gestational diabetes mellitus (GDM), the most common metabolic disorder of pregnancy, is a condition in which carbohydrate intolerance develops during pregnancy. The offspring of women with GDM are at an increased risk of macrosomia, neonatal hypoglycemia, and hyperbilirubinemia. The prevalence of GDM varies from 1 to 20%, and is rising worldwide, parallel to the increased prevalence of obesity and type 2 diabetes mellitus (T2DM) [1]. As reported previously, the prevalence of GDM in a population of pregnant women usually reflects the prevalence of T2DM in that particular population [2].

It is important to predict GDM early in pregnancy to enable early interventions that could prevent GDM and reduce adverse outcomes. Currently, there are no established guidelines for the prediction of GDM and no effective modalities for the prevention of its future development prior to actual diagnosis. According to existing literature, excessive body weight/obesity is the main risk factor for GDM [3]. Clinicians usually use body mass index (BMI) to assess maternal obesity during pregnancy. However, previous studies found that BMI may not be a good predictor for GDM around the first trimester [4, 5], due to its inability to reflect the accumulation and mass of the visceral adipose tissue (VAT) [6]. Recently, a promising echocardiographic parameter, epicardial adipose tissue (EAT), has emerged that could complement the use of BMI to assess risk for GDM. EAT is closely related to metabolic syndrome and diabetes [7, 8], and is an independent predictor of visceral adiposity, as measured by echocardiography [9]. This study aimed to investigate the association between the EAT thickness and GDM, and assess the efficacy of EAT thickness as a potential predictor for GDM at 16 weeks + 0 days to 19 weeks + 6 days before the diagnosis of GDM.

## Methods

### Study design, setting, and population

This prospective cohort study was conducted at the First Affiliated Hospital of China Medical University. All participants were admitted to our obstetric clinic between January 2014 and December 2018. All participants provided written informed consent and the study protocol was approved by the Medical Ethics Review Board of China Medical University (Shenyang, Liaoning, China).

### Criteria

Participants were eligible if they: (1) had a singleton pregnancy; (2) had their first pregnancy visit before 19 weeks + 6 days (gestational age was determined by ultrasound within 3 months of pregnancy confirmation); (3) signed the informed consent form and provided a complete medical history. Participants were not eligible if they had a history of diabetes (including GDM in previous pregnancies), hypertension, or cardiovascular diseases.

### Data collection between 16 weeks + 0 days and 19 weeks + 6 days

A range of anthropometric parameters were measured for each participant, including weight, height, heart rate, systolic blood pressure, and diastolic blood pressure; we also calculated the BMI [10]. At each visit, we also recorded the family history of diabetes as a parent or sibling may have been diagnosed as having diabetes in the interval since the previous visit [11].

A peripheral blood sample was collected from each participant before 19 weeks + 6 days; samples were collected in a vacutainer collection tube. We used the blood samples to determine the lipid profile of each participant, including triglyceride, total cholesterol, high-density lipoprotein cholesterol (HDL-C), and low-density lipoprotein cholesterol (LDL-C); these parameters were determined with an auto-analyzer (AU1000; Olympus, Tokyo, Japan).

Maternal echocardiography was also performed between 16 weeks + 0 days and 19 weeks + 6 days. All images were obtained using a Philips iE33 system (Philips Medical Systems, Bothell, WA, USA) with a 1.5/5 MHz phased array probe. Maternal EAT thickness was measured by echocardiography in the parasternal long-axis view at the level of the fold of Rindfleisch, between the free wall of the right ventricle and the anterior surface of the ascending aorta [12].

### Data collection between 24 weeks + 0 days and 28 weeks + 0 days

A two-hour 75-g oral glucose tolerance test (OGTT) was used to test for GDM; these tests were carried out after an overnight fast. The following morning, we tested the fasting plasma glucose concentration and then asked the participant to drink 250–300 mL of water containing 75 g of sugar. We then determined plasma glucose levels 1 hour and 2 hours later [13].

GDM was diagnosed according to the International Association of Diabetes and Pregnancy Study Groups [13]. A diagnosis of GDM was made when one or more of the test parameters equaled or exceeded the following cut points: fasting 92 mg/dL (5.1 mmol/L), 1-h 180 mg/dL (10.0 mmol/L), or 2-h 153 mg/dL (8.5 mmol/L).

### Follow-up of neonatal outcomes in GDM patients

Adverse neonatal outcomes were recorded after delivery, including large size for gestational age, neonatal hypoglycemia, admission to the neonatal intensive care unit (NICU), preterm delivery, and hyperbilirubinemia. Large size for gestational age was defined as the birth weight at or above the gestational age-specific 90th percentile [3]. Neonatal hypoglycemia was defined as a blood glucose concentration < 47 mg/dL (< 2.6 mmol/L) [14]. Preterm was defined as delivery before 37 weeks of pregnancy [15]. Hyperbilirubinemia was defined as a bilirubin level that, at any time after birth, exceeded the hour-specific phototherapy treatment threshold recommended in the American Academy of Pediatrics’ clinical practice guideline on the management of neonatal hyperbilirubinemia [16].

### Statistical analysis

Statistical analysis was performed using STATA version 14.0 software. Continuous parameters were expressed as the mean ± standard deviation. Non-normally distributed parameters were expressed as the median. Differences of normally distributed continuous parameters between groups were analyzed using the independent-samples *t*-test. Differences of non-normally distributed parameters between groups were analyzed using the Mann-Whitney U test. Differences of categorial parameters between groups were analyzed using Pearson’s chi-squared test. Odds ratio (OR) and 95% confidence intervals (CIs) of individual maternal factors, as potential predictors for GDM, were calculated using generalized linear models. Receiver-operating-characteristic (ROC) analysis was conducted to assess the discriminative capacity of any individual maternal factor for the prediction of GDM. A two-tailed *P* < 0.05 was used to define statistical significance.

## Results

### Baseline maternal characteristics

**Fig. 1:**
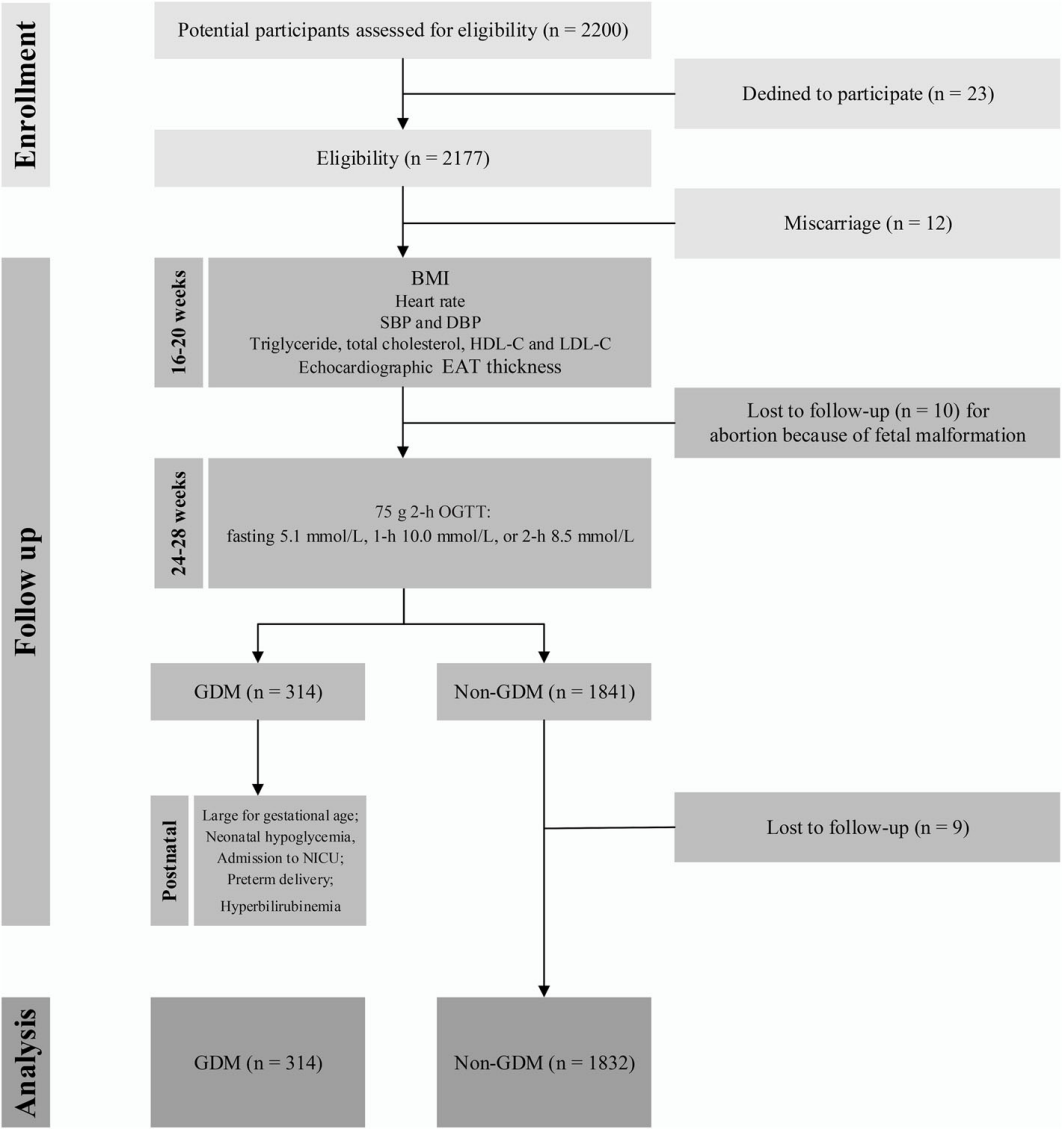
Study flow diagram. BMI, body mass index; DBP, diastolic blood pressure; EAT, epicardial adipose tissue; GDM, gestational diabetes mellitus; HDL-C, high-density lipoprotein cholesterol; LDL-C, low-density lipoprotein cholesterol; NICU, neonatal intensive care unit; OGTT, oral glucose tolerance test; SBP, systolic blood pressure

A total of 2146 mothers met our eligibility criteria and were included in the main analysis; 314 of these mothers had GDM and 1832 did not (Fig. 1). Table 1 shows the baseline clinical characteristics of the participants as compared between the GDM and control groups. There were significant differences between the two groups with respect to maternal age, BMI, lipid profiles (triglyceride, total cholesterol, and HDL-C), and EAT thickness (*P* = 0.018, *P* = 0.026, *P* = 0.018, *P* = 0.015, *P* = 0.007, *P* < 0.001, respectively). Overall, 47, 102, 113, and 52 participants received maternal echocardiography at 16–17, 17–18, 18–19, and 19–20 weeks of gestation in the GDM group. In the control group, 310, 628, 611, and 283 participants received maternal echocardiography at 16–17, 17–18, 18–19, and 19–20 weeks of gestation.

**Table 1:** Baseline characteristics of participants in GDM and control groups

	GDM (*n* = 314)	Control (*n* = 1832)	*P*
Maternal age (years)	32.10 ± 3.02	31.66 ± 3.01	0.018
Height (m)	1.63 ± 0.04	1.63 ± 0.05	0.106
Weight (kg)	60.42 ± 7.25	59.76 ± 7.11	0.128
BMI (kg/m^2^)	22.86 ± 3.07	22.46 ± 2.87	0.026
Gravida^a^	1	1	0.504
Parity	0	0	0.734
Family history of diabetes	21 (7%)	94 (5%)	0.258
Heart rate (beats/minute)	74.26 ± 8.04	73.36 ± 9.11	0.100
SBP (mmHg)	115.71 ± 5.71	115.20 ± 6.33	0.184
DBP (mmHg)	73.05 ± 6.00	72.87 ± 5.45	0.585
Triglyceride			
mg/dL	233.68 ± 61.59	224.81 ± 61.61	0.018
mmol/L	2.65 ± 0.70	2.55 ± 0.70	0.018
Total cholesterol			
mg/dL	240.92 ± 29.22	236.45 ± 30.19	0.015
mmol/L	6.26 ± 0.76	6.15 ± 0.78	0.015
HDL-C			
mg/dL	68.10 ± 9.01	69.31 ± 6.99	0.007
mmol/L	1.76 ± 0.23	1.79 ± 0.18	0.007
LDL-C			
mg/dL	129.37 ± 22.12	130.94 ± 21.82	0.239
mmol/L	3.36 ± 0.58	3.40 ± 0.57	0.239
EAT thickness (mm)	6.57 ± 0.79	5.49 ± 1.05	< 0.001

*BMI* Body mass index, *DBP* Diastolic blood pressure, *EAT* Epicardial adipose tissue, *GDM* Gestational diabetes mellitus, *HDL-C* High-density lipoprotein cholesterol, *LDL-C* Low-density lipoprotein cholesterol, *SBP* Systolic blood pressure. All the parameters (except for gravida, parity, and family history) were expressed as the mean ± standard deviation. Gravida and parity were expressed as the median

^a^Gravida describes the total number of confirmed pregnancies that a woman has had, regardless of the outcome

### Regression analysis for the presence of GDM

The results of all regression analyses are summarized in Table 2. Univariate regression analysis revealed that maternal age (OR = 1.05, 95% CI: 1.01–1.09), BMI (OR = 1.05, 95% CI: 1.01–1.09), triglyceride (OR = 1.23, 95% CI: 1.04–1.46), total cholesterol (OR = 1.21, 95% CI: 1.04–1.41), HDL-C (OR = 0.42, 95% CI: 0.22–0.79), and EAT thickness (OR = 2.92, 95% CI: 2.54–3.36) were significantly associated with the presence of GDM. Multivariate regression analysis further revealed that EAT thickness (OR = 2.87, 95% CI: 2.49–3.31) were significantly associated with the presence of GDM (*P <* 0.001).

**Table 2:** Results of univariate and multivariate regression analysis for the presence of GDM

	Univariate regression		Multivariate regression	
	OR (95%CI)	*P*	OR (95%CI)	*P*
Maternal age (years)	1.05 (1.01–1.09)	0.018	1.03 (0.98–1.07)	0.223
BMI (kg/m^2^)	1.05 (1.01–1.09)	0.026	1.03 (0.98–1.08)	0.230
Triglyceride (mg/dL)	1.23 (1.04–1.46)	0.018	1.16 (0.96–1.40)	0.130
Total cholesterol (mg/dL)	1.21 (1.04–1.41)	0.015	1.06 (0.90–1.26)	0.483
HDL-C (mg/dL)	0.42 (0.22–0.79)	0.007	0.42 (0.21–0.83)	0.011
EAT thickness (mm)	2.92 (2.54–3.36)	< 0.001	2.87 (2.49–3.31)	< 0.001

*BMI* Body mass index, *CI* Confidence interval, *EAT* Epicardial adipose tissue, *GDM* Gestational diabetes mellitus, *HDL-C* High-density lipoprotein cholesterol, *OR* Odds ratio

### ROC analysis

**Fig. 2:**
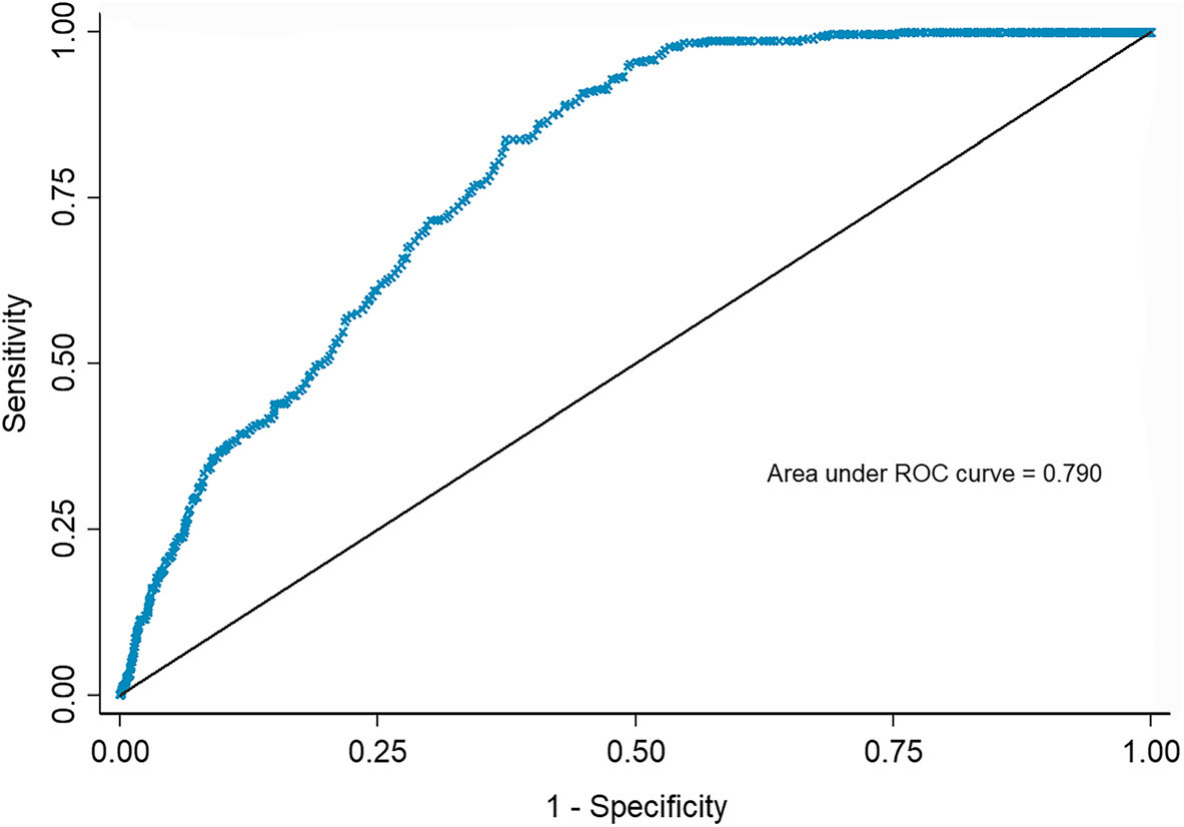
Receiver operating characteristic (ROC) curves for EAT thickness for the prediction of GDM. EAT, epicardial adipose tissue; GDM, gestational diabetes mellitus

ROC analysis was performed to verify whether EAT thickness could predict GDM. The ROC curve is shown in Fig. 2. The area under the curve was 0.790 (95% CI: 0.768–0.812). When the cutoff value was set to 5.49 mm, the sensitivity was 95.2% and the specificity was 50.5%.

### The association of EAT thickness with adverse outcomes in the GDM group

Table 3 shows that the EAT was significantly higher in patients with adverse outcomes when compared with the patients without adverse outcomes in the GDM group (*P <* 0.001). Regression analysis further revealed that EAT thickness was a significant risk factor for large size for gestational age (OR = 3.47, 95% CI: 2.29–5.26), neonatal hypoglycemia (OR = 3.10, 95% CI: 1.78–5.37), admission to NICU (OR = 4.38, 95% CI: 2.81–6.84), preterm delivery (OR = 4.67, 95% CI: 2.84–7.68), and hyperbilirubinemia (OR = 4.04, 95% CI: 2.24–7.28) (all *P <* 0.001). Overall, EAT thickness was a significant risk factor for adverse outcomes in GDM patients (OR = 8.28, 95% CI: 5.10–13.43, *P <* 0.001).

**Table 3:** Association of EAT thickness with adverse outcomes in the GDM group (*n* = 314)

	Cases with adverse outcomes	With adverse outcomes	Without adverse outcomes	P^*^	OR (95%CI)	*P*^*#*^
Total	91 (29%)	7.29 ± 0.66	6.27 ± 0.64	< 0.001	8.28 (5.10–13.43)	< 0.001
Large size for gestational age	54 (17%)	7.19 ± 0.80	6.43 ± 0.73	< 0.001	3.47 (2.29–5.26)	< 0.001
Neonatal hypoglycemia	23 (7%)	7.25 ± 0.73	6.51 ± 0.77	< 0.001	3.10 (1.78–5.37)	< 0.001
Admission to NICU	54 (17%)	7.28 ± 0.65	6.41 ± 0.74	< 0.001	4.38 (2.81–6.84)	< 0.001
Preterm delivery	41 (13%)	7.36 ± 0.53	6.44 ± 0.76	< 0.001	4.67 (2.84–7.68)	< 0.001
Hyperbilirubinemia	23 (7%)	7.39 ± 0.64	6.50 ± 0.77	< 0.001	4.04 (2.24–7.28)	< 0.001

*CI* Confidence interval, *EAT* Epicardial adipose tissue, *GDM* Gestational diabetes mellitus, *NICU* Neonatal intensive care unit, *OR* odds ratio

P^*^: *P*-value of independent-samples *t*-test

*P*^*#*^: *P*-value of univariate regression between epicardial adipose tissue thickness and adverse outcomes using generalized linear models

## Discussion

Possessing the ability to predict GDM as early as possible is a very important goal and has been pursued by researchers over many years as this could allow for early lifestyle changes and/or nutritional interventions to prevent GDM. Unfortunately, pregnancy is a complex and dynamic process, involving profound changes in energy and nutrient metabolism to sustain fetal development and growth, and to meet the requirements of labor and lactation. To date, although many risk factors for GDM have been identified (e.g., maternal age, obesity, and lipid profiles), no validated tool exists to predict the risk of GDM.

Clinicians usually use BMI, VAT, and subcutaneous adipose tissue (SAT), to assess obesity when trying to predict GDM. However, SAT is not a good predictor for GDM during the first trimester [17, 18]; neither is BMI [4, 5, 19]. Over recent years, several studies have revealed that VAT could be a good predictor for GDM in the first and second trimesters [18, 19]. It remains unclear whether EAT, as a special type of VAT, exerts a similar performance when screening for GDM.

EAT has a range of functions, including lipogenic capacity [20]. A number of researchers have focused on the adverse effects of EAT and confirmed this parameter as a marker of diabetic risk [8]. A previous study also revealed that EAT thickness was significantly higher in women with a history of GDM than controls [21]. Subsequently, two cross-sectional studies found a difference in EAT thickness when comparing between GDM and control groups during the second trimester [22, 23]. In these two previous studies, EAT thickness was measured at 24–28 gestational weeks (GW) at the time of GDM diagnosis. However, this is not the appropriate time for clinicians to make early interventions to prevent GDM. Therefore, we designed this study to identify whether EAT can act as a potential predictor during the early second trimester.

EAT thickness can be measured by echocardiography during pregnancy. This measurement has been proven to be both accurate and reproducible [12]. Abnormal levels of blood sugar in GDM patients usually appear after 20 GW [24]. Hence, we measured EAT thickness at 16 weeks + 0 days to 19 weeks + 6 days, a time point at which hyperglycemia is generally not present. This measurement was therefore taken 1 to 2 months prior to diagnosis, and would therefore be highly beneficial in that it could identify patients who have a risk of GDM and provide clinicians with sufficient time to develop appropriate treatment strategies.

Our analysis revealed that EAT thickness was significantly increased in the GDM group compared to the control group when measured between 16 weeks + 0 days and 19 weeks + 6 days. Furthermore, higher EAT thickness was significantly associated with adverse outcomes in GDM patients. We propose that there are two mechanisms that might be responsible for the increased EAT thickness in GDM patients. Firstly, the higher levels of retinol-binding protein 4 and lower levels of adiponectin secreted by adipose tissue, including EAT, prior to 16 GW [25, 26], could cause insulin resistance (IR), the main mechanisms underlying GDM [27]. Secondly, during the first-second trimester, EAT releases higher levels of pro-inflammatory adipokines (RBP4, hs-CRP, fatty acid-binding protein-4, leptin, and visfatin), and lower levels of anti-inflammatory adipokines (omentin-1 and adiponectin); these may participate in the chronic low-grade state of inflammation that has previously been confirmed to be associated with GDM [26, 28].

Pregnancy can be viewed as a cardiovascular stress test in that the development of certain complications has the potential to reveal a woman’s susceptibility for future vascular or metabolic disease [29]. A previous study showed that GDM patients who had an increased EAT thickness during pregnancy were associated with subclinical atherosclerosis 6 years postpartum [21, 30]. The association between EAT and postpartum cardiovascular diseases therefore needs to be addressed further in future research. We aim to set up a new study, with a longer follow-up duration, to determine whether patients at a high-risk of GDM, as identified by EAT, are associated with T2DM and cardiovascular diseases. This type of study will allow us to implement preventive measures for this particular population.

### Limitations

There are some limitations to our study that need to be considered. For example, all of our participants were Asian women. It is possible that race may be an important consideration when analyzing the relationship between EAT and the risk of disease [31]. Furthermore, we did not analyze data relating to inflammatory biomarkers; such data may be beneficial with regards to the efficacy of the EAT thickness model during the early second trimester.

## Conclusion

Collectively, our findings indicate that echocardiographic EAT thickness is positively and significantly associated with GDM risk and adverse outcomes related to GDM. Echocardiographic EAT has the potential to be a predictor for GDM prior to actual clinical diagnosis.

## Data Availability

The datasets used and/or analyzed during the current study are available from the corresponding author on reasonable request.

## Abbreviations

BMI: Body mass index
CIs: Confidence intervals
GDM: Gestational diabetes mellitus
GW: Gestational week
HDL-C: High-density lipoprotein cholesterol
IR: Insulin resistance
LDL-C: Low-density lipoprotein cholesterol
OR: Odds ratio
T2DM: Type 2 diabetes mellitus
VAT: Visceral adipose tissue.
